# Combination GLP-1RA and Low-Dose IL-2 Modulates Peripheral Immune Activation and Attenuates CNS Inflammatory Transcript Signatures In Vivo

**DOI:** 10.3390/ijms27177855

**Published:** 2026-09-02

**Authors:** Aaron D. Thome, Jinghong Wang, Alireza Faridar, Weihua Zhao, Valerie Saetzler, David R. Beers, Stanley H. Appel

**Affiliations:** Department of Neurology, Houston Methodist Neurological Institute, Houston Methodist Research Institute, Houston Methodist Hospital, Houston, TX 77030, USA

**Keywords:** neuroimmunology, neuroinflammation, regulatory T cells, myeloid cells, GLP-1RA, low-dose IL-2, immunomodulation

## Abstract

Immune dysregulation characterized by persistent myeloid activation and associated impairment of regulatory T cell (Treg) function contributes to inflammatory signaling across peripheral and central compartments in neurodegenerative diseases. We evaluated whether combining a glucagon-like peptide-1 receptor agonist (GLP-1RA; semaglutide) with low-dose interleukin-2 (LD-IL2) could modulate myeloid-associated transcript expression and enhance Treg-associated regulatory transcripts in a subacute lipopolysaccharide (LPS)-induced model of systemic and CNS inflammation. Mice received LPS once daily for 5 days, while GLP-1RA, LD-IL2, or combination treatment was initiated 24 h after LPS onset and continued daily. Splenic immune populations were quantified, and transcript expressions were assessed in magnetically enriched CD11b^+^ myeloid cells and CD4^+^CD25^+^ Tregs, as well as in cortex and hippocampus. As monotherapies, GLP-1RA reduced myeloid expansion with modest modulation of pro-inflammatory and anti-inflammatory myeloid transcripts, whereas LD-IL2 selectively enhanced Treg numbers and increased transcripts associated with Treg stability and suppressive regulation, including *Il2ra* (CD25), *Foxp3*, *Ctla4*, *Ikzf2* (HELIOS), *Entpd1* (CD39), and *Nt5e* (CD73). Combination treatment significantly reduced LPS-induced myeloid *Il6*, *Il1b*, and *Tnf* expression and increased *Arg1* expression. Combination treatment further enhanced Treg-associated *Il2ra* (CD25), *Tgfb1*, and *Ctla4* expression relative to monotherapies. In cortical and hippocampal tissues, combination treatment produced more robust modulation of inflammatory transcripts compared with effects observed with monotherapies, including reductions in *Il6* and *Il1b* and increases in *Cd163* and *Mrc1* (CD206) expression. Together, these findings demonstrate coordinated and complementary changes in peripheral immune-cell populations and myeloid inflammatory and Treg-associated regulatory transcripts and warrant further evaluation of this combination in inflammation-driven neurodegenerative disease.

## 1. Introduction

Neurodegenerative diseases such as Parkinson’s disease (PD), Alzheimer’s disease (AD), and amyotrophic lateral sclerosis (ALS) are characterized by neuroinflammation, systemic immune dysfunction, and progressive neuronal loss. Despite major advances in understanding neurodegenerative disease biology, current therapeutic strategies remain largely symptomatic, offering limited impact on neuronal loss and clinical decline. A growing body of evidence has established neuroinflammation and systemic immune dysregulation as central drivers of disease progression rather than bystander responses to neuronal injury [1,2,3,4,5,6,7,8]. Heightened peripheral immune activation, increased pro-inflammatory cytokine signaling, and impaired regulatory immune mechanisms can amplify microglial reactivity, hinder inflammatory resolution, and destabilize neuronal homeostasis, thereby fueling a self-perpetuating cycle of inflammation and neurodegeneration. Among the most consistently observed immune alterations are persistent activation and dysfunction of peripheral and central nervous system (CNS) myeloid populations, including peripheral monocytes/macrophages and CNS-resident microglia [2,6,8,9], together with impaired regulatory T cell (Treg) number and function, both of which have been associated with disease severity and progression [10,11,12,13,14].

Therapeutically targeting disease-associated immune dysregulation therefore represents a promising approach to restore immune balance and interrupt inflammatory processes that sustain and accelerate neurodegeneration. Chronic myeloid inflammatory signaling can amplify peripheral cytokine responses and CNS immune activation, while emerging evidence suggests that chronic myeloid dysfunction may also directly impair Treg-mediated regulation and inflammatory resolution [15,16,17,18,19,20]. These interconnected abnormalities provide a rationale for simultaneously attenuating inflammatory myeloid activity and reinforcing Treg-mediated immune regulation. Treg-enhancing approaches and glucagon-like peptide-1 receptor agonists (GLP-1RAs) have each independently demonstrated anti-inflammatory and neuroprotective effects in preclinical and clinical settings. However, each approach primarily addresses a different component of immune dysfunction, and their combined therapeutic potential in vivo remains largely unexplored.

Glucagon-like peptide-1 receptor agonists (GLP-1RAs), such as exenatide, liraglutide, and semaglutide, have demonstrated both systemic and CNS immunomodulatory effects and are being evaluated as promising anti-inflammatory therapies in preclinical models and clinical trials [21,22,23]. Experimental studies indicate that activation of the GLP-1 receptor attenuates NF-κB and MAPK signaling in macrophages and microglia, reducing the production of IL-6, IL-1β, and TNF, while promoting alternative (M2-like) polarization and the expression of tissue-restorative mediators. In preclinical models of PD and AD, GLP-1RAs have been shown to reduce dopaminergic neuron loss, mitigate amyloid and tau pathology, and improve synaptic and cognitive outcomes across multiple neurodegenerative disease models [24,25,26,27,28,29,30,31,32,33,34,35,36]. While clinical translation of GLP-1RA’s anti-inflammatory and neuroprotective effects has shown encouraging results in AD and PD trials, their overall disease-modifying effects are mixed across the trials [37,38,39,40,41,42,43,44,45]. In parallel, to address the Treg dysfunction characteristic of neurodegenerative disease, low-dose interleukin-2 (LD-IL2) has emerged as an immunomodulatory strategy that selectively expands and stabilizes Tregs without broadly stimulating effector immunity. The LD-IL2 approach activates the high-affinity IL-2 receptor (IL2RA/CD25) which is preferentially expressed on Tregs, leading to enhanced immunomodulatory markers and suppressive function [46,47]. Preclinically, LD-IL2 has been shown to enhance Treg numbers and suppressive function, reduce microglial activation and neuroinflammatory signaling, and confer beneficial neuroprotection in models of inflammatory, autoimmune, and neurodegenerative diseases [48,49,50,51,52,53]. Clinically, LD-IL2 has been shown to expand circulating Treg numbers, increase suppressive function, and normalize inflammatory cytokine profiles in several autoimmune and neurodegenerative disease indications [53,54,55,56,57,58,59].

Although Treg-enhancing and myeloid-targeted therapies have demonstrated immunomodulatory effects individually, emerging evidence suggests that these pathways are mechanistically interdependent. Chronic activation of pro-inflammatory myeloid cells can sustain peripheral and central cytokine signaling, contributing to inflammatory microenvironments in which pro-inflammatory cytokines can destabilize FOXP3 expression and impair Treg suppressive capacity [60,61,62,63]. In this context, Treg-enhancing strategies alone may encounter a biological ceiling, as persistent immune activation continues to impose functional constraints on regulatory stability. Conversely, suppression of inflammatory myeloid signaling without restoration of Treg function may be insufficient to reestablish durable immune tolerance. These observations support an approach in which effective immune recalibration in chronic inflammatory states may require simultaneous attenuation of pathogenic myeloid activation and reinforcement of Treg stability and function, particularly when the goal is to surpass the partial immunomodulation achieved by single-modality approaches. Supporting this rationale, our previous in vitro studies using human peripheral immune cells demonstrated that combined GLP-1RA and LD-IL2 treatment enhanced Treg suppression of pro-inflammatory myeloid activity and responder T-cell proliferation, reduced inflammatory myeloid transcripts, and increased Treg-associated regulatory and survival transcripts [64]. These prior findings provide the foundation for evaluating whether complementary modulation of these immune pathways could also be observed in vivo.

To evaluate the therapeutic potential of combining semaglutide, a GLP-1RA, with LD-IL2 in a focused in vivo proof-of-concept setting, we utilized a subacute low-dose lipopolysaccharide (LPS) mouse model, which induces sustained peripheral immune activation and measurable CNS neuroinflammation without the acute toxicity associated with high-dose paradigms. This model recapitulates key features relevant to neurodegenerative disease, including elevated pro-inflammatory signaling, myeloid activation, and compromised immune regulation, providing a reproducible platform for assessing therapeutic immunomodulation. Notably, repeated low-dose LPS exposure at the levels used here has been shown to induce Alzheimer’s disease-like pathology, including cognitive impairment, as well as Parkinson’s disease-associated neuroinflammation and α-synuclein accumulation [65,66,67,68,69,70,71,72,73,74,75]. These characteristics of the LPS model provide a reproducible in vivo framework for focused proof-of-concept evaluation and immune-modulation signal detection across peripheral and CNS inflammatory mechanisms relevant to neurodegenerative disease.

## 2. Results

### 2.1. GLP-1RA Reduces Peripheral Myeloid Expansion and LD-IL2 Enhances Tregs, with Combination Treatment Producing Coordinated Immune Modulation in an LPS-Induced Inflammatory Mouse Model

**Figure 1:**
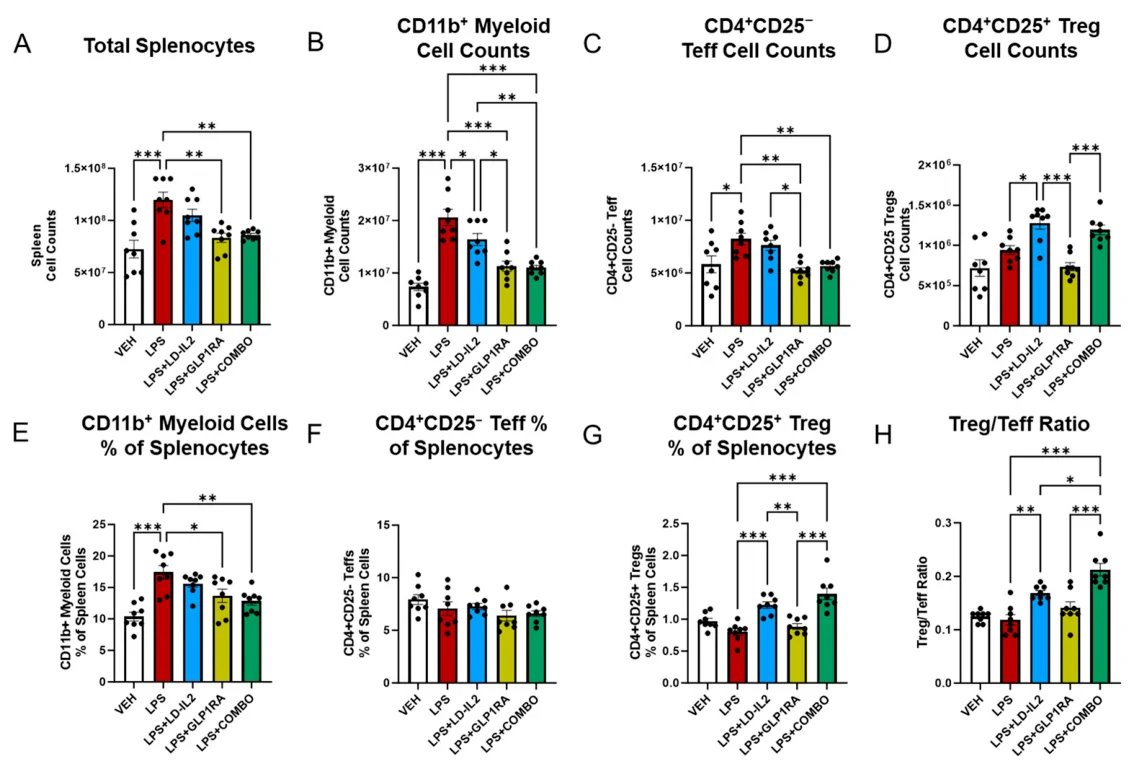
Splenic immune-cell populations in the LPS-induced peripheral and CNS inflammation model. Absolute cell numbers and frequencies are presented as mean ± SEM, with *n* = 8–10 animals per group and each data point representing one animal. CD11b^+^ myeloid cells, CD4^+^CD25^−^ T cells, and CD4^+^CD25^+^ Tregs were enriched by Miltenyi magnetic column separation. Absolute counts represent estimated whole-spleen values adjusted for parallel isolation workflows. (**A**) Total splenocytes; (**B**) CD11b^+^ myeloid cells; (**C**) CD4^+^CD25^−^ T cells; (**D**) CD4^+^CD25^+^ Tregs; (**E**–**G**) corresponding percentages of total splenocytes; and (**H**) Treg/CD4^+^CD25^−^ T-cell ratio. Statistical analysis was performed using ordinary one-way ANOVA followed by Tukey’s multiple-comparisons test. Significance is indicated as *p* < 0.05 (*), *p* < 0.01 (**), and *p* < 0.001 (***). Selected pairwise comparisons are displayed; complete statistical results are provided in the Supplemental Data S1.

CD11b^+^ myeloid cells, CD4^+^CD25^−^ Teff cells, and CD4^+^CD25^+^ Treg cells were enriched from mouse spleens following five days of 0.5 mg/kg LPS administration. LPS significantly increased total splenocyte numbers from 7.25 × 10^7^ in vehicle-treated mice to 1.20 × 10^8^, whereas GLP-1RA monotherapy and LD-IL2 + GLP-1RA combination treatment reduced splenocyte counts toward baseline to 8.34 × 10^7^ and 8.60 × 10^7^, respectively (Figure 1A). A similar pattern was observed in the CD11b^+^ myeloid compartment, with LPS increasing myeloid-cell numbers to 2.06 × 10^7^. LD-IL2 monotherapy produced a partial but significant reduction to 1.64 × 10^7^, while GLP-1RA monotherapy and combination treatment further reduced myeloid-cell numbers to 1.13 × 10^7^ and 1.10 × 10^7^, respectively (Figure 1B). GLP-1RA monotherapy and combination treatment also reduced LPS-associated increases in total Teff cells. In contrast, LD-IL2 significantly increased Treg numbers to 1.28 × 10^6^ compared with 9.41 × 10^5^ following LPS alone, while combination treatment maintained elevated Treg numbers at 1.20 × 10^6^ (Figure 1C,D).

Normalization to total splenocyte counts confirmed these treatment-associated shifts in immune-cell composition. LPS increased the proportion of CD11b^+^ myeloid cells from 10.38% to 17.40%, whereas GLP-1RA monotherapy and combination treatment reduced myeloid frequency to 13.68% and 12.83%, respectively (Figure 1E). Teff frequency remained comparatively stable across groups at approximately 6–7% (Figure 1F). In contrast, Treg frequency increased from 0.80% following LPS alone to 1.22% with LD-IL2 and 1.40% with combination treatment (Figure 1G). Consistent with these changes, the Treg/Teff ratio was highest following combination treatment at 0.21 compared with 0.11 following LPS alone and 0.17 with LD-IL2 monotherapy (Figure 1H). Collectively, these findings demonstrate coordinated modulation of peripheral immune-cell populations, with GLP-1RA primarily attenuating LPS-associated myeloid expansion and LD-IL2 preferentially enhancing the Treg compartment.

### 2.2. Combination GLP-1RA and LD-IL2 Modulates Pro- and Anti-Inflammatory-Associated Transcripts in Splenic Myeloid Cells

**Figure 2:**
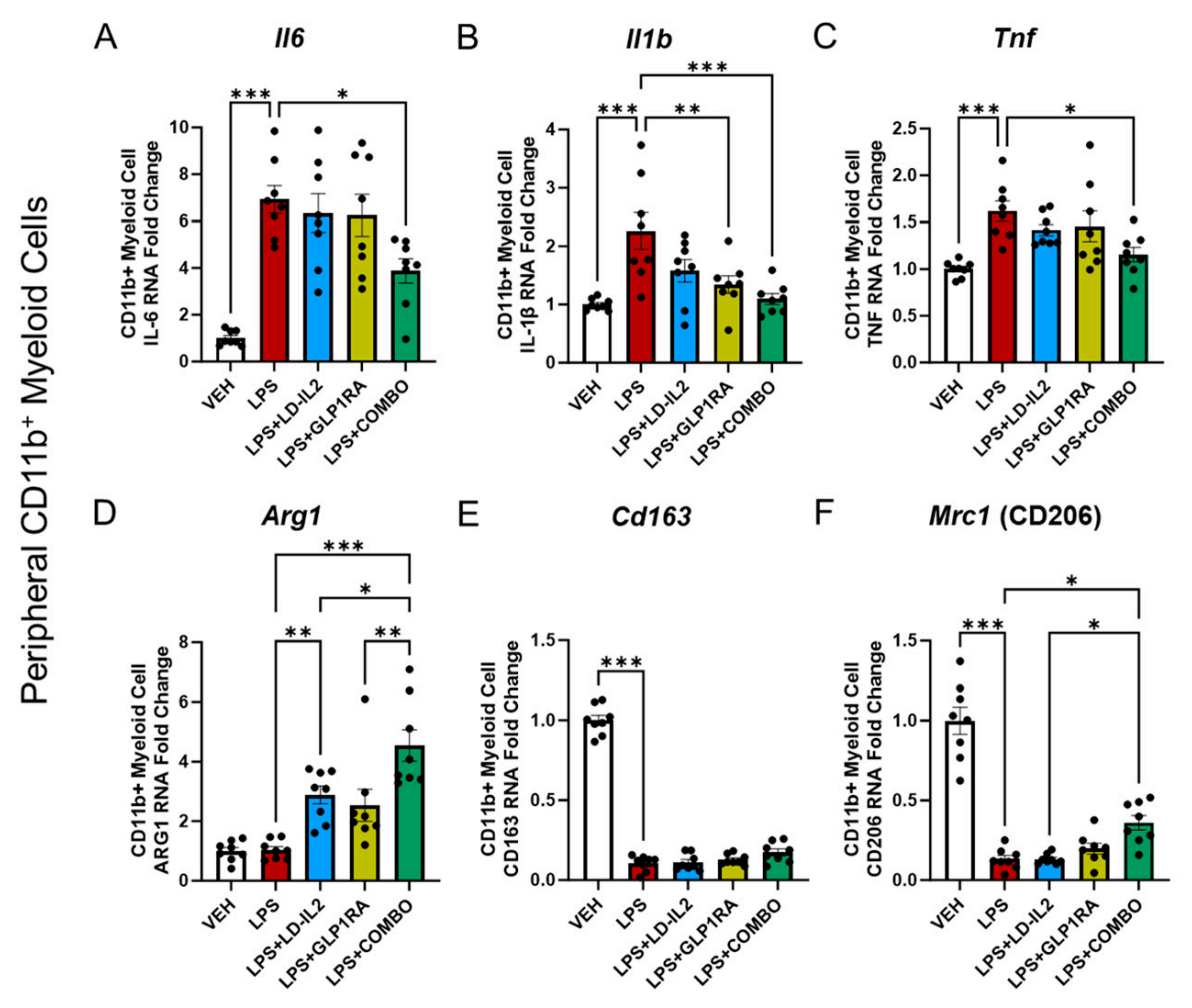
Inflammatory transcript expression in splenic CD11b^+^ myeloid cells in the LPS-induced peripheral and CNS inflammation model. Transcript levels are presented as mean ± SEM, with *n* = 8–10 animals per group and each data point representing one animal. CD11b^+^ myeloid cells were enriched from spleens by Miltenyi magnetic column separation. Pro-inflammatory transcripts include (**A**) *Il6*, (**B**) *Il1b*, and (**C**) *Tnf*; anti-inflammatory-associated transcripts include (**D**) *Arg1*, (**E**) *Cd163*, and (**F**) *Mrc1* (CD206). Expression was quantified by RT-qPCR, normalized to *A*ctb, and reported as fold change relative to vehicle controls. Statistical analysis was performed using ordinary one-way ANOVA followed by Tukey’s multiple-comparisons test. Significance is indicated as *p* < 0.05 (*), *p* < 0.01 (**), and *p* < 0.001 (***). Selected pairwise comparisons are displayed; complete statistical results are provided in the Supplemental Data S1.

CD11b^+^ myeloid cells were enriched from spleens and evaluated for transcripts associated with pro-inflammatory and anti-inflammatory myeloid programs. LPS administration markedly increased the pro-inflammatory transcripts interleukin-6 (*Il6*), interleukin-1 beta (*Il1b*), and tumor necrosis factor (*Tnf*) to 6.92-fold, 2.63-fold, and 1.62-fold relative to vehicle, respectively. Although LD-IL2 or GLP-1RA monotherapy produced only modest reductions in *Il6* expression, combination LD-IL2 + GLP-1RA treatment decreased *Il6* from 6.92-fold to 3.88-fold relative to vehicle (Figure 2A). GLP-1RA monotherapy significantly reduced *Il1b* expression relative to LPS, while combination treatment reduced *Il1b* from 2.63-fold to 1.10-fold (Figure 2B). Combination treatment also reduced *Tnf* expression from 1.62-fold to 0.90-fold compared with LPS alone (Figure 2C).

Among transcripts associated with anti-inflammatory myeloid programs, LPS markedly reduced *Cd163* and mannose receptor C-type 1 (*Mrc1*/CD206) expression, whereas arginase 1 (*Arg1*) expression remained near vehicle levels. LD-IL2 significantly increased *Arg1* expression relative to LPS, while combination treatment produced the highest *Arg1* expression at 4.55-fold compared with 1.04-fold following LPS alone (Figure 2D). *Cd163* and *Mrc1* (CD206) expression were reduced by LPS to 0.10-fold and 0.13-fold, respectively. *Cd163* remained comparatively low across the treatment groups, whereas combination treatment significantly increased *Mrc1* (CD206) expression to 0.36-fold (Figure 2E,F). Overall, combination treatment produced broader modulation across pro-inflammatory and anti-inflammatory-associated myeloid transcripts than either monotherapy alone.

### 2.3. LD-IL2 Enhances Peripheral Treg-Associated Regulatory Transcripts, with Additive Modulation Following Combination Treatment

**Figure 3:**
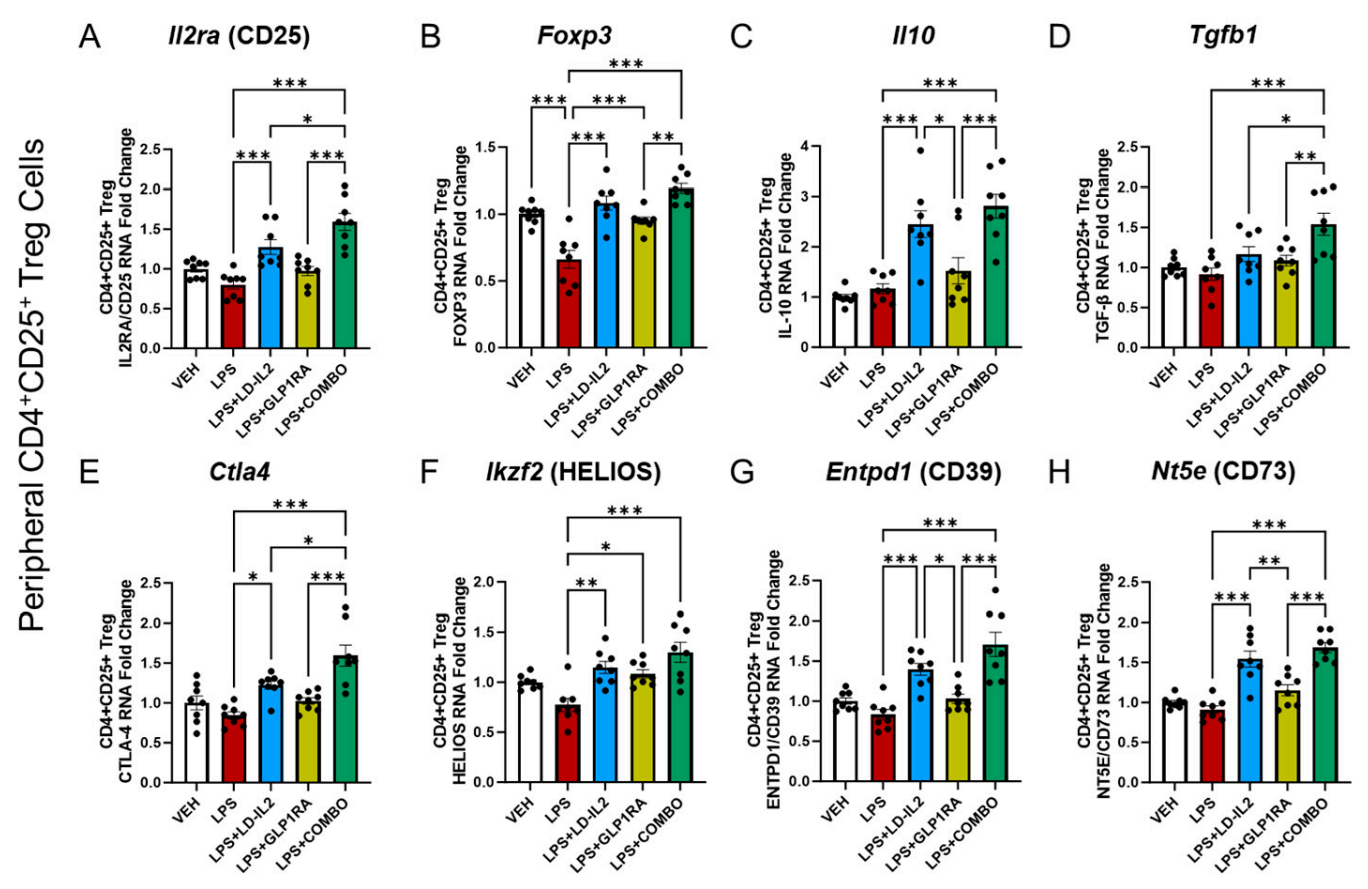
Treg-associated transcript expression in splenic CD4^+^CD25^+^ Tregs. Transcript levels are presented as mean ± SEM, with *n* = 8–10 animals per group and each data point representing one animal. CD4^+^CD25^+^ Tregs were enriched from spleens by magnetic column separation. Transcripts associated with Treg identity, stability, and suppressive regulation include (**A**) *Il2ra* (CD25), (**B**) *Foxp3*, (**C**) *Il10*, (**D**) *Tgfb1*, (**E**) *Ctla4*, (**F**) *Ikzf2* (HELIOS), (**G**) *Entpd1* (CD39), and (**H**) *Nt5e* (CD73). Expression was quantified by RT-qPCR, normalized to *Actb*, and reported as fold change relative to vehicle controls. Statistical analysis was performed using ordinary one-way ANOVA followed by Tukey’s multiple-comparisons test. Significance is indicated as *p* < 0.05 (*), *p* < 0.01 (**), and *p* < 0.001 (***). Selected pairwise comparisons are displayed; complete statistical results are provided in the Supplemental Data S1.

CD4^+^CD25^+^ Tregs were enriched from mouse spleens following five days of 0.5 mg/kg LPS administration with test article treatments. LPS alone did not significantly alter Treg cell numbers or frequency. Among the Treg-associated transcripts evaluated, LPS significantly reduced forkhead box P3 (*Foxp3*) expression to 0.66-fold relative to vehicle, whereas the other measured transcripts were not significantly reduced by LPS. Interleukin-2 receptor alpha (*Il2ra*/CD25) expression was 0.80-fold following LPS treatment and increased to 1.28-fold with LD-IL2 monotherapy, while GLP-1RA monotherapy had minimal effect (0.97-fold). Combination LD-IL2 + GLP-1RA treatment produced the highest *Il2ra* (CD25) expression at 1.59-fold (Figure 3A). *Foxp3* expression, which was significantly reduced by LPS, increased with both monotherapies and combination treatment, reaching 1.08-fold with LD-IL2 and 1.20-fold with combination treatment (Figure 3B). LD-IL2 also increased interleukin-10 (*Il10*) expression to 2.45-fold, with combination treatment reaching 2.81-fold, while transforming growth factor beta 1 (*Tgfb1*) expression increased to 1.54-fold with combination treatment (Figure 3C,D).

We next evaluated transcripts associated with Treg stability and suppressive regulation, including cytotoxic T-lymphocyte-associated protein 4 (*Ctla4*), IKAROS family zinc finger 2 (*Ikzf2*/HELIOS), ectonucleoside triphosphate diphosphohydrolase 1 (*Entpd1*/CD39), and 5′-nucleotidase ecto (*Nt5e*/CD73). LD-IL2 increased *Ctla4* expression from 0.89-fold following LPS to 1.23-fold, while combination treatment increased *Ctla4* to 1.59-fold (Figure 3E). *Ikzf2* (HELIOS) expression increased with both LD-IL2 and GLP-1RA monotherapy relative to LPS, with combination treatment reaching 1.31-fold (Figure 3F). LD-IL2 also increased *Entpd1* (CD39) expression from 0.87-fold to 1.40-fold and *Nt5e* (CD73) expression from 0.94-fold to 1.55-fold, with expression reaching 1.71-fold and 1.69-fold, respectively, following combination treatment (Figure 3G,H). In the absence of inflammatory challenge, combination-only treatment also increased multiple Treg-associated regulatory transcripts, as reported in the Supplemental Data S1. Notably, across the Treg-associated transcript panel, the monotherapy response was predominantly associated with LD-IL2, whereas GLP-1RA alone produced comparatively limited changes. Combination treatment maintained the LD-IL2-associated response while producing additional significant increases in selected transcripts, including *Il2ra* (CD25), *Tgfb1*, and *Ctla4*.

### 2.4. GLP-1RA and LD-IL2 Modulate Cortical and Hippocampal Inflammatory Transcripts, with Enhanced Effects Following Combination Treatment

**Figure 4:**
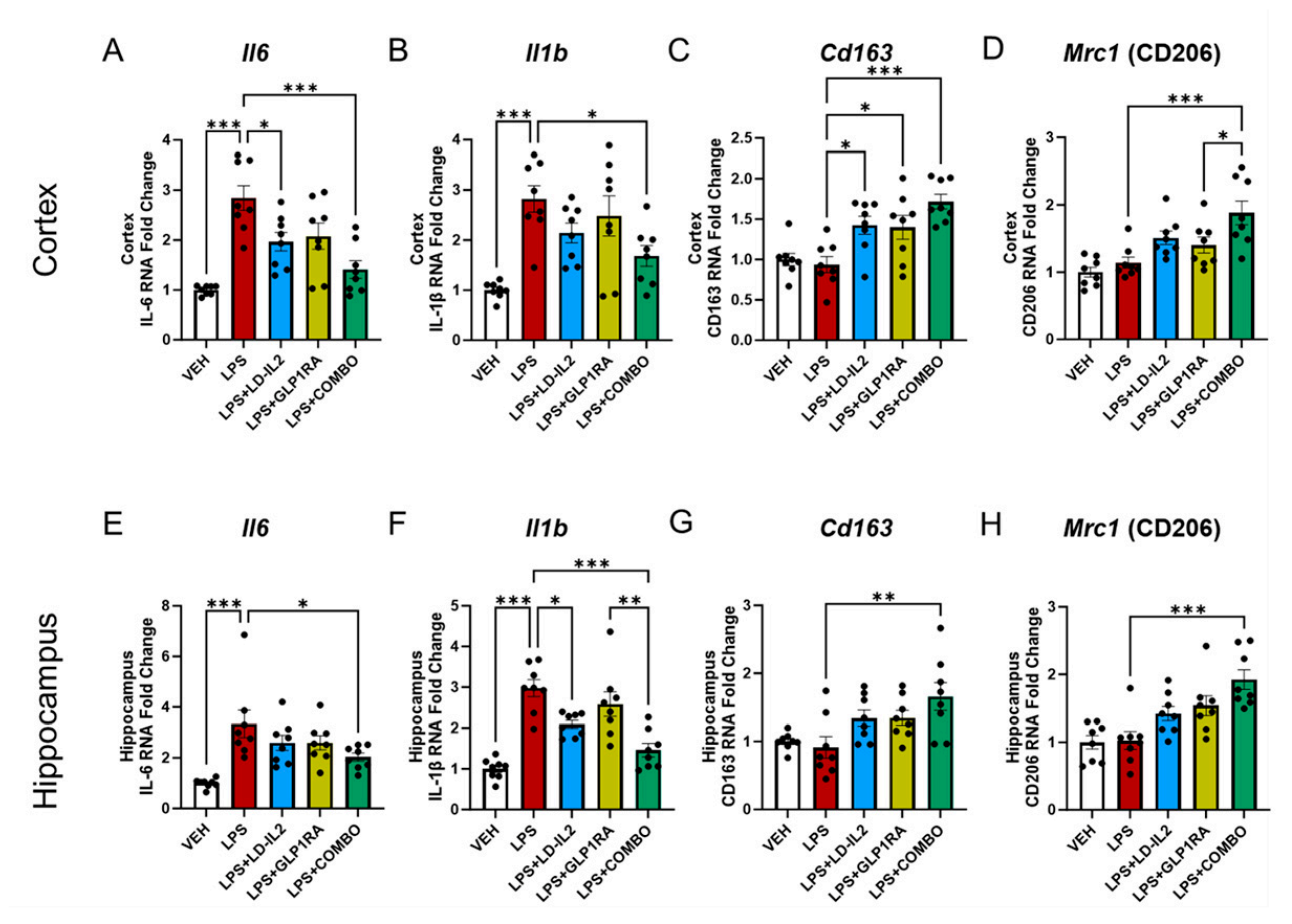
Inflammatory transcript expression in cortical and hippocampal tissues. Transcript levels are presented as mean ± SEM, with *n* = 8-10 animals per group and each data point representing one animal. Cortex panels show (**A**) *Il6*, (**B**) *Il1b*, (**C**) *Cd163*, and (**D**) *Mrc1* (CD206); hippocampus panels show (**E**) *Il6*, (**F**) *Il1b*, (**G**) *Cd163*, and (**H**) *Mrc1* (CD206). Expression was quantified by RT-qPCR using RNA isolated from microdissected brain tissue, normalized to *Actb*, and reported as fold change relative to vehicle controls. Statistical analysis was performed using ordinary one-way ANOVA followed by Tukey’s multiple-comparisons test. Significance is indicated as *p* < 0.05 (*), *p* < 0.01 (**), and *p* < 0.001 (***). Selected pairwise comparisons are displayed; complete statistical results are provided in the Supplemental Data S1.

To determine whether treatment was associated with changes in inflammatory transcriptional signatures within CNS tissues, we examined inflammation-related transcript expression in the cortex and hippocampus. In the cortex, LPS increased *Il6* expression to 2.85-fold relative to vehicle. LD-IL2 significantly reduced *Il6* expression to 2.03-fold, while combination LD-IL2 + GLP-1RA treatment produced a greater reduction to 1.41-fold (Figure 4A). LPS also increased *Il1b* expression to 2.82-fold, with combination treatment significantly reducing expression to 1.68-fold (Figure 4B). Among anti-inflammatory-associated transcripts, *Cd163* expression was significantly increased by both LD-IL2 and GLP-1RA monotherapies relative to LPS and reached 1.72-fold following combination treatment (Figure 4C). Combination treatment also significantly increased *Mrc1* (CD206) expression from 1.14-fold following LPS to 1.89-fold (Figure 4D).

In the hippocampus, LPS increased *Il6* expression to 3.40-fold, while combination treatment significantly reduced expression to 2.07-fold (Figure 4E). LPS also increased *Il1b* to 2.98-fold; LD-IL2 significantly reduced expression to 2.09-fold, while combination treatment produced a larger reduction to 1.46-fold (Figure 4F). Combination treatment additionally significantly increased *Cd163* expression from 0.93-fold following LPS to 1.66-fold and *Mrc1* (CD206) from 1.02-fold to 1.93-fold (Figure 4G,H). Overall, monotherapies produced significant effects on selected cortical and hippocampal transcripts, whereas combination treatment produced the broadest pattern of significant modulation across the CNS transcript panel. These findings are interpreted as changes in inflammatory transcriptional signatures within CNS tissue rather than evidence of a specific CNS myeloid-cell phenotype.

## 3. Discussion

In this study, combined glucagon-like peptide-1 receptor agonist (GLP-1RA) and low-dose interleukin-2 (LD-IL2) treatment produced complementary cellular and transcriptional effects across peripheral immune populations and CNS tissues in a LPS-induced inflammatory mouse model. GLP-1RA treatment primarily reduced splenic CD11b^+^ myeloid-cell expansion and modulated inflammatory myeloid transcripts, whereas LD-IL2 increased CD4^+^CD25^+^ Treg numbers and enhanced expression of transcripts associated with Treg identity, stability, and suppressive regulation, including *Il2ra* (CD25), *Foxp3*, *Ctla4*, *Ikzf2* (HELIOS), *Entpd1* (CD39), and *Nt5e* (CD73). Combination treatment preserved these modality-specific effects while producing a broader pattern of immune modulation, including significant reductions in myeloid *Il6* and *Tnf* expression, increased *Arg1* expression, and enhanced Treg-associated *Il2ra* (CD25), *Tgfb1*, and *Ctla4* expression relative to monotherapies. These coordinated peripheral effects were accompanied by reduced *Il6* and *Il1b* expression and increased *Cd163* and *Mrc1* (CD206) expression in cortical and hippocampal tissues. Collectively, these findings support a complementary framework in which concurrent modulation of myeloid inflammatory transcripts and reinforcement of Treg-associated regulatory transcripts can produce broader immune modulation than either strategy alone.

The observed transcriptional changes are consistent with the established immunomodulatory actions of GLP-1RAs and LD-IL2. GLP-1 receptor activation has been shown to suppress inflammatory signaling in macrophages and microglia, including NF-κB- and MAPK-associated pathways, thereby reducing expression or production of IL-6, IL-1β, and TNF and promoting transcriptional and metabolic programs associated with less inflammatory myeloid states [21,22,23,34,35,36]. Consistent with these mechanisms, GLP-1RA treatment in the present study reduced LPS-induced splenic myeloid expansion and modulated pro-inflammatory and anti-inflammatory myeloid transcripts. These findings support GLP-1RA-associated modulation of myeloid-cell abundance and inflammatory transcript responses within the LPS-driven inflammatory environment; however, the present design does not establish whether these effects resulted from direct GLP-1 receptor signaling in myeloid cells or from indirect systemic mechanisms.

LD-IL2 acts primarily through preferential engagement of the high-affinity IL-2 receptor expressed by Tregs, with downstream JAK1/JAK3-STAT5 signaling supporting Treg expansion, survival, and maintenance of regulatory transcriptional programs [48,49,50,51,53,76,77,78]. In the present study, LD-IL2 increased splenic CD4^+^CD25^+^ Treg numbers and enhanced multiple transcripts associated with Treg stability and suppressive capacity, including *Foxp3*, *Il2ra* (CD25), *Ctla4*, *Ikzf2* (HELIOS), *Entpd1* (CD39), and *Nt5e* (CD73). The combination with GLP-1RA produced additional increases in selected Treg-associated transcripts, particularly *Il2ra* (CD25), *Tgfb1*, and *Ctla4*. These findings are consistent with reinforcement of Treg-associated regulatory programs but should not be interpreted as direct evidence of restored suppressive function because functional Treg assays were not performed.

In the present model, LPS engagement of TLR4 activates downstream MyD88-dependent and MyD88-independent signaling pathways that converge on NF-κB and other inflammatory transcriptional programs, thereby driving robust myeloid activation and inflammatory cytokine production across peripheral and CNS compartments [79,80,81]. The biological rationale for combining these modalities is based on the interdependence of inflammatory myeloid activity and regulatory immune function. Persistent myeloid activation can sustain production of inflammatory mediators and contribute to pro-inflammatory microenvironments in which cytokine signaling can impair Treg stability and suppressive activity, thereby constraining Treg-mediated immune regulation and inflammatory resolution [60,61,62,63]. Conversely, inadequate Treg-mediated control can permit continued myeloid activation and inflammatory amplification, while effective Treg regulation can directly restrain inflammatory myeloid programs [82,83]. Targeting either pathway alone may therefore leave the complementary component of this inflammatory circuit insufficiently controlled. In this context, GLP-1RA-mediated attenuation of myeloid inflammatory signaling may reduce inflammatory signals that contribute to Treg dysfunction, thereby creating a more permissive environment for LD-IL2-mediated Treg expansion and reinforcement of regulatory programs, while enhanced Treg-associated regulation may provide additional restraint on ongoing myeloid activation and inflammatory propagation. Although the predominant effects of GLP-1RA and LD-IL2 are conceptualized here as myeloid-directed and Treg-directed, respectively, these activities may not be entirely compartment-specific. GLP-1R expression has been reported in Tregs, and prior studies suggest that GLP-1R signaling may directly influence Treg stability and survival [64,84,85,86]. Conversely, myeloid populations can express IL2RA/CD25 under inflammatory conditions, and limited evidence suggests that IL-2 signaling may directly influence myeloid activation states [87,88,89]. In addition, LD-IL2-mediated enhancement of Treg regulation may secondarily restrain myeloid inflammatory activity. Whether these direct or indirect cross-lineage effects contribute to the broader combination response observed here remains to be determined. A conceptually similar combination strategy has been evaluated clinically in ALS using CTLA4-Ig together with LD-IL2, based on the hypothesis that persistent pro-inflammatory myeloid activity can contribute to Treg dysfunction and limit the durability of Treg-enhancing therapies. In that phase 1 study, combined treatment increased Treg suppressive function and was accompanied by reductions in biomarkers of inflammation and oxidative stress, providing clinical precedent for pairing attenuation of inflammatory immune activity with reinforcement of Treg-mediated regulation [58]. The broader combination-associated transcriptional profile observed across peripheral immune cells and CNS tissues is consistent with this complementary framework, although the direct cellular interactions underlying these effects were not evaluated in the present study.

Importantly, GLP-1RA and LD-IL2 treatment was initiated 24 h after the first LPS administration, demonstrating treatment-associated effects after inflammatory induction had begun. Combination treatment was associated with effects across both isolated peripheral immune populations and bulk cortical and hippocampal tissues. In the CNS, the combination significantly reduced *Il6* and *Il1b* expression while increasing *Cd163* and *Mrc1* (CD206) expression, whereas monotherapies produced limited changes across these endpoints. Because the CNS analyses were performed on bulk tissue, the cellular sources of these transcripts cannot be determined. The observed changes may reflect altered activity of resident microglia, infiltrating myeloid cells, or other CNS-associated cell populations and should therefore be interpreted as modulation of CNS inflammatory transcriptional signatures rather than direct evidence of microglial polarization.

The combination produced a broader pattern of statistically significant and directional effects than either monotherapy alone. Although formal pharmacologic interaction testing was not performed, these results are consistent with potential additive immunomodulatory effects. They do not establish pharmacologic synergy, nor do they demonstrate that combination treatment was statistically superior to both monotherapies for every endpoint. Rather, the data support coordinated engagement of distinct immune compartments, with GLP-1RA primarily modulating myeloid abundance and inflammatory transcript responses and LD-IL2 primarily increasing Treg abundance and reinforcing Treg-associated regulatory transcripts. Future studies incorporating factorial interaction analyses and larger cohorts will be required to formally distinguish additive from synergistic effects.

These findings also have translational relevance because GLP-1RAs and LD-IL2 have each undergone substantial clinical evaluation as individual modalities and act through distinct immunomodulatory mechanisms. GLP-1RAs are being investigated in neurodegenerative disorders based on their metabolic, anti-inflammatory, and neuroprotective properties, while LD-IL2 has been evaluated as a strategy to selectively expand Tregs in autoimmune and neurodegenerative conditions. More broadly, LD-IL2 represents one of several therapeutic strategies aimed at reinforcing regulatory immunity, alongside adoptive Treg transfer, ex vivo expanded Treg therapies, and other regulatory CD4^+^ T-cell approaches [11,90,91,92]. These cell-based strategies provide complementary evidence that enhancement of regulatory immune activity can restrain pathogenic inflammation, whereas LD-IL2 offers a pharmacologic means of expanding and supporting endogenous Treg populations in vivo. The present combination therefore differs conceptually from Treg-focused monotherapy by pairing reinforcement of endogenous regulatory immunity with attenuation of inflammatory myeloid signaling through GLP-1RA. Because both agents were administered systemically, effects in immune compartments beyond the spleen, including intestinal and mesenteric immune populations, could also contribute indirectly to peripheral or CNS inflammatory responses through the gut-brain axis. LD-IL2 has been shown to expand Tregs within intestinal and mesenteric compartments [93], while semaglutide can modulate intestinal barrier-associated pathways and gut microbial composition in preclinical models [94]. Systemic LPS exposure can also disrupt intestinal epithelial integrity and increase intestinal permeability [95]. However, intestinal immune populations, gut barrier integrity, and microbiome-associated changes were not evaluated in the present study, and any contribution of gut-associated immune modulation remains speculative. The present findings provide a rationale for evaluating whether their complementary activities can generate broader and more durable immune modulation than either modality alone. However, the safety, pharmacodynamic interactions, and immunologic consequences of combined administration will require direct evaluation before clinical translation.

Several limitations should be considered. The subacute LPS model reproduces systemic and CNS inflammatory activation but does not recapitulate the chronic, progressive, and disease-specific pathology of neurodegenerative disorders. The short treatment period also precluded assessment of long-term durability, cumulative immunologic effects, neuronal injury, behavioral outcomes, or disease modification. The study was designed with limited scope as a focused evaluation of immune-cell and CNS tissue transcriptional responses. Consequently, protein-level validation, cytokine measurements, functional Treg suppression assays, histologic characterization, and spatial or single-cell profiling were not performed. Accordingly, changes in transcript expression cannot be assumed to reflect corresponding changes in protein abundance, cytokine production, or immune-cell function. In addition, post-isolation purity of the magnetically enriched immune-cell populations was not independently confirmed by flow cytometry, and the bulk CNS analyses prevent attribution of transcriptional changes to specific cell populations. The present findings should therefore be interpreted as coordinated changes in inflammatory and regulatory transcriptional signatures rather than definitive evidence of functional immune restoration or neuroprotection.

Future studies should evaluate this combination in chronic and disease-relevant neurodegenerative models and incorporate longitudinal behavioral outcomes, protein-level inflammatory measurements, histologic assessment of neuronal and glial pathology, functional Treg assays, and cell-resolved immune profiling. These approaches will be necessary to determine the cellular specificity, durability, and functional consequences of the observed immune modulation and to establish whether the potential additive effects translate into meaningful neuroprotection.

Overall, combined GLP-1RA and LD-IL2 treatment produced complementary modulation of peripheral immune-cell populations and myeloid- and Treg-associated transcripts across peripheral immune cells and CNS tissues. The broader transcriptional response observed with combination treatment supports further mechanistic evaluation of concurrent myeloid-modulating and Treg-enhancing immunomodulation in inflammation-driven neurodegenerative disease.

## 4. Materials and Methods

### 4.1. Animals and Experimental Design

Male C57BL/6J mice (10–12 weeks of age, 20–28 g; The Jackson Laboratory, Bar Harbor, ME, USA) were used in this study. Animals were group-housed under controlled temperature and humidity with a 12 h light/dark cycle and ad libitum access to food and water. Mice were acclimated for at least 72 h before study initiation and randomly assigned to experimental groups with body weights balanced across groups.

Experimental groups included vehicle control, LPS alone, LPS + LD-IL2, LPS + GLP-1RA, LPS + LD-IL2 + GLP-1RA, and LD-IL2 + GLP-1RA without LPS. Each experimental group included 8–10 animals, with the individual mouse serving as the biological experimental unit. The exact number of animals included in each analysis is reported in the corresponding figures and Supplemental Data S1. The experimental timeline and treatment paradigm are summarized in Supplemental Figure S1. Group sizes of 8–10 animals were informed by previously published comparable systemic LPS studies assessing peripheral and CNS inflammatory endpoints [71,96,97] and by pilot studies confirming measurable inflammatory transcript activation in peripheral and CNS tissues using the selected LPS dosing paradigm. A formal a priori power calculation was not performed because the study was designed as a focused proof-of-concept evaluation incorporating multiple cellular and transcriptional endpoints rather than a single predefined primary endpoint.

All animal procedures were approved by the Houston Methodist Institutional Animal Care and Use Committee (IACUC; protocol IS00008830) and conducted in accordance with institutional guidelines and the National Institutes of Health Guide for the Care and Use of Laboratory Animals.

### 4.2. LPS-Induced Inflammation and Treatment Administration

Systemic and central nervous system inflammation was induced using lipopolysaccharide from *Escherichia coli* O111:B4 (LPS; Sigma-Aldrich, St. Louis, MO, USA). LPS was reconstituted in sterile saline and administered intraperitoneally at 0.5 mg/kg once daily for five consecutive days. This dosing paradigm was selected based on previously reported models demonstrating sustained peripheral and CNS inflammatory responses [65,72,98].

Semaglutide, a glucagon-like peptide-1 receptor agonist (GLP-1RA; MedChemExpress, Monmouth Junction, NJ, USA), was prepared according to the manufacturer’s instructions and administered intraperitoneally at 25 nmol/kg once daily. The dose, route, and frequency were selected based on previously established preclinical paradigms demonstrating anti-inflammatory activity in mice [99,100,101,102].

Clinical-grade human interleukin-2 (Coya Therapeutics, Inc., Houston, TX, USA), formulated at 1,000,000 IU/mL under Good Manufacturing Practice conditions, was administered intraperitoneally at a fixed low dose of 30,000 IU per mouse once daily. This dose was selected from published murine low-dose IL-2 paradigms using recombinant human IL-2 at approximately 25,000–50,000 IU per mouse, which have demonstrated biological activity in murine T cells and expansion or enhancement of Treg populations [50,76,103,104,105,106]. The use of clinical-grade human IL-2 also provides translational relevance by evaluating the human therapeutic cytokine preparation rather than a murine IL-2 surrogate.

LPS was administered on study Days 1–5. GLP-1RA, low-dose IL-2 (LD-IL2), or their combination was initiated 24 h after the first LPS administration and administered on Days 2–5, for a total of four treatment doses. This post-induction treatment schedule was designed to evaluate immunomodulatory activity after inflammatory signaling had been initiated [75,96,97,107]. LPS and administration volumes were calculated according to individual body weight, whereas LD-IL2 was administered at a fixed dose per mouse. Animals were weighed and monitored daily for clinical signs of systemic inflammation and adherence to humane endpoints. All injections were performed at consistent times by the same personnel to minimize procedural variability.

### 4.3. Euthanasia and Tissue Collection

Mice were euthanized on Day 5 within 24 h of the final LPS administration. Animals were deeply anesthetized with 5% inhaled isoflurane (Fluriso, VetOne/MWI Veterinary Supply Co., Boise, ID, USA) in an induction chamber, and adequate anesthesia was confirmed by absence of the pedal-withdrawal reflex. Blood was collected by cardiac puncture, followed by exsanguination under deep anesthesia. For brain collection, animals were decapitated under anesthesia immediately after exsanguination to facilitate rapid preservation of CNS tissue. Death was confirmed by cessation of respiration and cardiac activity. Euthanasia procedures were performed in accordance with the American Veterinary Medical Association Guidelines for the Euthanasia of Animals.

Spleens and brains were collected immediately after euthanasia. Cortical and hippocampal regions were microdissected, flash-frozen on dry ice, and stored at −80 °C until RNA isolation. Spleens were transferred into cold RPMI-1640 medium (Gibco, Thermo Fisher Scientific, Waltham, MA, USA) and processed immediately for immune-cell isolation.

### 4.4. Splenocyte Preparation and Immune-Cell Isolation

Spleens were mechanically dissociated through a 70 µm cell strainer (Falcon, Corning Incorporated, Corning, NY, USA) into cold RPMI-1640 medium to generate single-cell suspensions. Red blood cells were removed using ammonium–chloride–potassium lysis buffer (Gibco, Thermo Fisher Scientific, Waltham, MA, USA), followed by washing in ice-cold buffer. Total splenocyte numbers and viability were determined by hemocytometer counting with trypan blue (Corning Incorporated, Corning, NY, USA) exclusion before magnetic isolation.

Splenocytes from each animal were divided evenly for parallel isolation of CD11b^+^ myeloid cells and CD4^+^ T-cell populations. CD11b^+^ myeloid cells were isolated by positive selection using CD11b MicroBeads (Miltenyi Biotec, Bergisch Gladbach, Germany). CD4^+^CD25^+^ Tregs and CD4^+^CD25^−^ T cells were isolated using the CD4^+^CD25^+^ Regulatory T Cell Isolation Kit (Miltenyi Biotec, Bergisch Gladbach, Germany). This procedure involved negative selection of non-CD4^+^ cells followed by positive selection of CD25^+^ cells from the enriched CD4^+^ population. Magnetic separations were performed according to the manufacturer’s recommended procedures and bead-to-cell ratios. This magnetic enrichment approach has previously been reported to achieve ≥90% purity for murine CD4^+^CD25^+^ Treg populations [108,109,110,111,112]; however, post-enrichment purity was not independently assessed in the present study.

Recovered cell populations were quantified by hemocytometer counting with trypan blue exclusion. Because each splenocyte suspension was divided for parallel isolation workflows, recovered cell numbers were adjusted according to the fraction of the original suspension used to estimate whole-spleen cell counts for figure reporting. Isolated cells were immediately lysed for RNA extraction. Cell yields were prioritized for the predefined transcriptional analyses; therefore, post-isolation population purity was not independently confirmed by flow cytometry in this experiment.

### 4.5. RNA Purification and RT-qPCR

Total RNA was isolated from magnetically enriched immune-cell populations and microdissected cortical and hippocampal tissues using TRIzol reagent (Invitrogen, Thermo Fisher Scientific, Waltham, MA, USA). RNA was subsequently purified using the Direct-zol RNA MiniPrep Plus Kit (Zymo Research, Irvine, CA, USA) according to the manufacturer’s instructions. Immune-cell populations were lysed directly in TRIzol immediately after magnetic enrichment. When immediate extraction was not possible, lysates were stored at −80 °C. Frozen cortical and hippocampal tissues were transferred directly into TRIzol and homogenized during RNA extraction.

RNA concentration and purity were assessed using a NanoDrop ND-1000 spectrophotometer (Thermo Fisher Scientific, Waltham, MA, USA). Reverse transcription and quantitative PCR were performed using the iTaq Universal SYBR Green One-Step Kit with commercially available, prevalidated gene-specific primer assays on a CFX Connect™ Real-Time PCR Detection System using CFX Manager™ software, version 3.1 (all Bio-Rad Laboratories, Hercules, CA, USA), according to the manufacturer’s protocols. Reactions were performed in technical replicates, and replicate cycle-threshold values were averaged before analysis. Relative gene expression was calculated using the $2^{-\Delta \Delta Ct}$ method, with *Actb* as the reference gene and the vehicle group as the calibrator.

### 4.6. Statistical Analysis

Statistical analyses were performed using GraphPad Prism version 10.1.2 (GraphPad Software, Boston, MA, USA). The individual animal was considered the experimental unit. Data are presented as mean ± standard error of the mean unless otherwise indicated. Differences among vehicle, LPS, monotherapy, and combination-treatment groups were evaluated using ordinary one-way analysis of variance followed by Tukey’s multiple-comparisons test. All tests were two-sided, and adjusted p-values were used to determine statistical significance.

Statistical significance is indicated as: *p* < 0.05 (*), *p* < 0.01 (**), and *p* < 0.001 (***). Selected comparisons relevant to the predefined study objectives are displayed in the figures for clarity. Exact sample sizes and complete pairwise statistical outputs from the ANOVA models are provided in the corresponding figure legends and Supplemental Data S1.

## 5. Conclusions

Combined GLP-1RA and LD-IL2 produced complementary immunomodulatory effects across peripheral immune populations and CNS tissues in an LPS-induced inflammatory model. GLP-1RA primarily attenuated myeloid expansion and inflammatory transcriptional responses, whereas LD-IL2 increased Treg numbers and reinforced transcriptional programs associated with Treg stability and suppressive regulation. The broader response observed with combination treatment is consistent with potential additive modulation of these interconnected immune pathways. These findings provide a biological rationale for further evaluation of combined myeloid-modulating and Treg-enhancing therapies in chronic, disease-relevant models of neuroinflammation and neurodegeneration.

## Data Availability

The data supporting the findings of this study are included within the article and its Supplementary Materials. Additional raw data are available from the corresponding authors upon reasonable request.

## Supplementary Materials

The following supporting information can be downloaded at: https://www.mdpi.com/article/10.3390/ijms27177855/s1.

## Abbreviations

The following abbreviations are used in this manuscript:

Abbreviation	Definition
ACTB	Actin beta
AD	Alzheimer’s disease
ALS	Amyotrophic lateral sclerosis
ANOVA	Analysis of variance
ARG1	Arginase 1
AVMA	American Veterinary Medical Association
CNS	Central nervous system
CTLA-4	Cytotoxic T-lymphocyte-associated protein 4
ENTPD1/CD39	Ectonucleoside triphosphate diphosphohydrolase 1/cluster of differentiation 39
FOXP3	Forkhead box P3
GLP-1RA	Glucagon-like peptide-1 receptor agonist
IACUC	Institutional Animal Care and Use Committee
IKZF2/HELIOS	Ikaros family zinc finger 2/HELIOS
IL-1β	Interleukin-1 beta
IL-2	Interleukin-2
IL-6	Interleukin-6
IL-10	Interleukin-10
IL2RA/CD25	Interleukin-2 receptor subunit alpha/cluster of differentiation 25
IU	International units
LD-IL2	Low-dose interleukin-2
LPS	Lipopolysaccharide
MAPK	Mitogen-activated protein kinase
MRC1/CD206	Mannose receptor C-type 1/cluster of differentiation 206
NF-κB	Nuclear factor kappa B
NIH	National Institutes of Health
NT5E/CD73	5′-Nucleotidase ecto/cluster of differentiation 73
PD	Parkinson’s disease
RNA	Ribonucleic acid
RT-qPCR	Reverse transcription quantitative polymerase chain reaction
SEM	Standard error of the mean
Teff	Effector T cell
TGF-β	Transforming growth factor beta
TNF	Tumor necrosis factor
Treg	Regulatory T cell
