# Supplementary material for: Combination GLP-1RA and Low-Dose IL-2 Modulates Peripheral Immune Activation and Attenuates CNS Inflammatory Transcript Signatures In Vivo
Source: Int J Mol Sci. 2026 Sep 2;27(17):7855. doi: 10.3390/ijms27177855 (PMC13566286; doi:10.3390/ijms27177855)
Supplement: Supplementary file 1 [file ijms-27-07855-s001.zip › Supplementary Figure S1.pdf]

Day	-4	-3	-2	-1	0	1	2	3	4	5
LPS 0.5 mg/kg IP					X	X	X	X	X	
LD-IL2 (30,000 IU) IP						X	X	X	X	
GLP-1RA (25 nmol/kg) IP						X	X	X	X	
Combo LD-IL2 + GLP-1RA (2X IP)						X	X	X	X	
Animal Welfare Check				X	X	X	X	X	X	
Animal Weight				X	X	X	X	X	X	X
Sacrifice Endpoint										X

Supplemental Figure S1. Experimental timeline and treatment paradigm for the subacute LPS-induced inflammation model. Mice received intraperitoneal LPS (0.5 mg/kg) once daily on Days 1–5. GLP-1RA (25 nmol/kg), LD-IL2 (30,000 IU/mouse), or their combination was administered intraperitoneally once daily on Days 2–5, beginning 24 h after the first LPS dose. Mice were euthanized within 24 h of the final LPS administration for collection of spleen, cortex, and hippocampus. Experimental groups included vehicle, LPS alone, LPS + LD-IL2, LPS + GLP-1RA, LPS + LD-IL2 + GLP-1RA, and LD-IL2 + GLP-1RA without LPS. Each experimental group included n = 8-10 animals.
